# Comparing Robot-Assisted Laparoscopic Pyeloplasty vs. Laparoscopic Pyeloplasty in Infants Aged 12 Months or Less

**DOI:** 10.3389/fped.2021.647139

**Published:** 2021-06-14

**Authors:** Yuenshan Sammi Wong, Kristine Kit Yi Pang, Yuk Him Tam

**Affiliations:** Division of Paediatric Surgery and Paediatric Urology, Department of Surgery, Prince of Wales Hospital, The Chinese University of Hong Kong, Shatin, Hong Kong

**Keywords:** ureteropelvic junction obstruction, infant, standard laparoscopy, pyeloplasty in infants, robot assisted laparoscopy

## Abstract

**Objective:** To investigate the outcomes of minimally invasive approach to infants with ureteropelvic junction (UPJ) obstruction by comparing the two surgical modalities of robot-assisted laparoscopic pyeloplasty (RALP) and laparoscopic pyeloplasty (LP).

**Methods:** We conducted a retrospective review of all consecutive infants aged ≤12 months who underwent either LP or RALP in a single institution over the period of 2008–Jul 2020. We included primary pyeloplasty cases that were performed by or under the supervision of the same surgeon.

**Results:** Forty-six infants (LP = 22; RALP = 24) were included with medians of age and body weight at 6 months (2–12months) and 8.0 kg (5.4–10 kg), respectively. There was no difference between the two groups in the patients' demographics and pre-operative characteristics. All infants underwent LP or RALP successfully without conversion to open surgery. None had intraoperative complications. Operative time (OT) was 242 min (SD = 59) in LP, compared with 225 min (SD = 39) of RALP (*p* = 0.25). Linear regression analysis showed a significant trend of decrease in OT with increasing case experience of RALP(*p* = 0.005). No difference was noted in the post-operative analgesic requirement. RALP was associated with a shorter hospital length of stay than LP (3 vs. 3.8 days; *p* = 0.009). 4/22(18%) LP and 3/24(13%) RALP developed post-operative complications (*p* = 0.59), mostly minor and stent-related. The success rates were 20/22 (91%) in LP and 23/24 (96%) in RALP (*p* = 0.49).

**Conclusions:** Pyeloplasty by minimally invasive approach is safe and effective in the infant population. RALP may have superiority over LP in infants with its faster recovery and a more manageable learning curve to acquire the skills.

## Introduction

Previous studies of meta-analysis have shown that both laparoscopic pyeloplasty (LP) and robot-assisted laparoscopic pyeloplasty (RALP) are viable options to treat ureteropelvic junction (UPJ) obstruction in children with the benefits of shorter hospital stay and decreased morbidity while maintaining a success rate comparable to open pyeloplasty (OP) (1–3). The contemporary evidence of performing pyeloplasty by minimally invasive approach in the infant population, however, are less robust than in older children as there are few comparative studies ever published (4–6).

The expanding interest in minimally invasive pyeloplasty in children is mainly brought by the momentum of the robotic technology. National trends study in the United States between 2003 and 2015 showed that LP decreased annually by a rate of 12% while RALP grew by 29% annually (7). By 2015, RALP accounted for 40% of total cases and comprised 84% of cases among adolescents (7). A big contrast, however, was noted in the infant population in which 85% of cases were OP while RALP and LP accounted for 10 and 5%, respectively in 2015 (7). Adoption of minimally invasive approach in infants has been slow due to the perceived technical challenges associated with the anatomical and physiological constraints of infants and the high success rate by OP (5, 6, 8). Infants were excluded in some of the comparative studies (9, 10).

Our institution has adopted the minimally invasive approach for correction of UPJ obstruction across the entire pediatric age groups for two decades (11). LP had been our standard until Jan 2014 when it was replaced by RALP. In this study we aimed to compare the outcomes of the two minimally invasive modalities in infants. We hypothesized that RALP has superiority over LP in infants.

## Materials and Methods

After getting the approval of the clinical research ethics committee of our institution, we retrospectively reviewed the medical records of all consecutive infants aged 12 months or less who underwent either LP or RALP for UPJ obstruction in our institution over the period of 2008–July 2020. We included those primary pyeloplasties which were performed by or under the supervision of the senior author of this study using standardized surgical techniques, and similar pre- and post-operative management protocols. Re-operative pyeloplasty was excluded. All the LP cases were recruited before Jan 2014, and since then all the infant pyeloplasties had been performed by the robotic approach.

Before surgery, all patients had ultrasound (US) and MAG3 scan which showed Society of Fetal Urology (SFU) grade 3 or 4 hydronephrosis, and obstructed drainage with diuretic *t*-half > 20 min of the affected kidney. Indications for surgery included progressive worsening of hydronephrosis in serial US, drop in split renal function <45% in the initial or repeat MAG3, giant hydronephrosis with mass effect requiring percutaneous nephrostomy (PCN) decompression in neonatal period, and urosepsis.

All patients had double-J stent inserted at the time of pyeloplasty and was removed in 3–4 weeks. Routine post-operative evaluation included both US and MAG3 scan in 2–3 months after double-J stent removal. US was then repeated in 6 months and then yearly if the initial post-operative investigations suggested successful pyeloplasty. Success of surgery was defined by absence of repeat intervention plus 1 or more of the following radiological criteria: (i) resolution of hydronephrosis with anteroposterior diameter (APD) of renal pelvis <10 mm in US, (ii) improved drainage in MAG3 scan with diuretic *t*-half <20 min, (iii) reduction in hydronephrosis with stable split renal function in MAG3 scan.

We collected data on patients' demographics, clinical characteristics at baseline, post-operative radiological findings, operative details, complications, analgesic requirement, length of hospital stay (LOS), and follow-up period. Operative time (OT) was defined by the time interval from the first skin incision to completion of wound closure. Post-operative complications were graded according to the Clavien classification (12).

**Figure 1:**
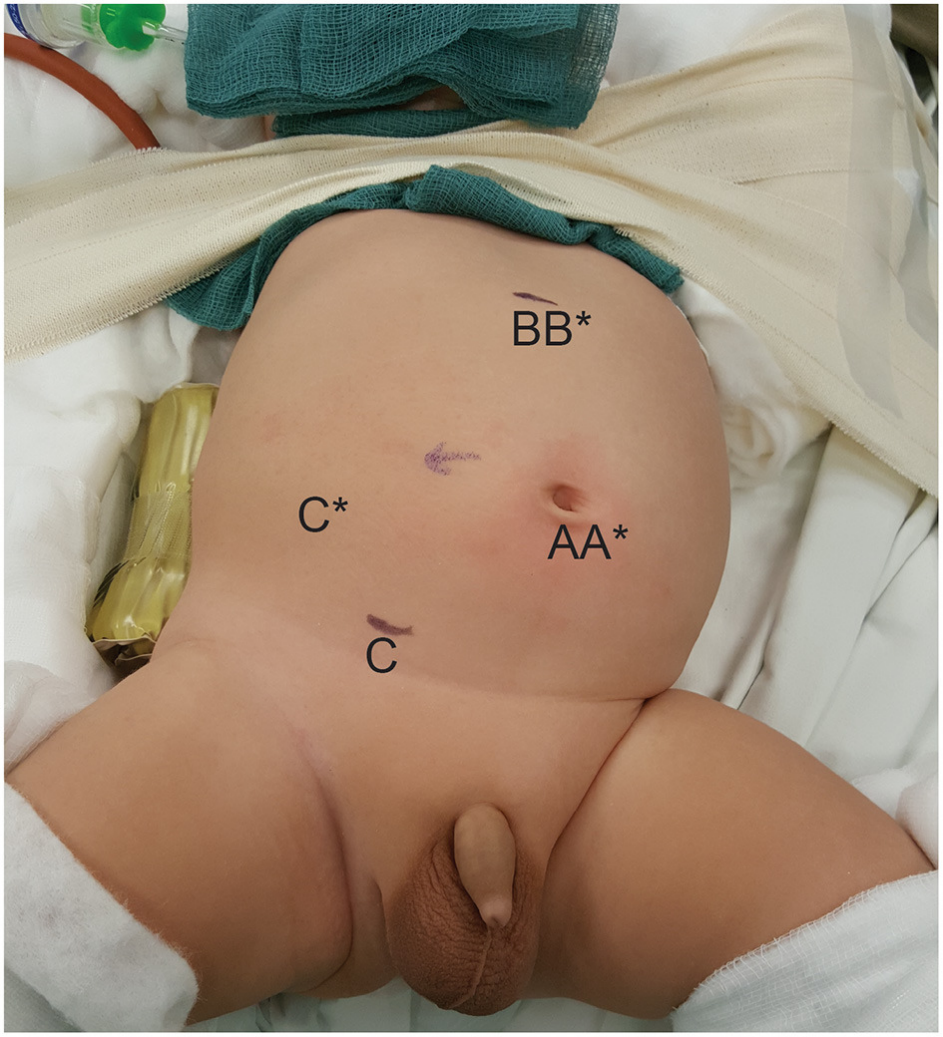
Positions of the ports in a 3-month-old infant who underwent right-sided RALP. A, B, and C, positions of the ports in RALP; A*, B*, and C*, positions of the ports if the procedure were LP.

We have previously described our technique of LP and RALP (13). Transperitoneal approach was used in both LP and RALP. The surgical steps of the two approaches were almost identical. Only three ports were used in both approaches with a single transabdominal hitching suture to lift and stabilize the renal pelvis. No accessory port was used in RALP. We used 3-mm instruments in LP and 8-mm instruments in RALP. The initial cases of RALP were performed by the da Vinci S model (Intuitive Surgical, Sunnyvale, CA) which was subsequently replaced by the Xi model. In RALP, we placed a purse-string suture to tighten the musculofascial defect around the camera port at the umbilicus and the suture was further tied onto the short rubber latex tube placed around the port. The two working ports were placed at sub-xiphoid and suprainguinal region just lateral to the inferior epigastric vessels under the laparoscopic view (Figure 1). A double-J stent was routinely inserted by antegrade method over guidewire introduced transabdominally. Cystoscopy would be used if difficulty was encountered in passing the double-J stent into bladder by antegrade method. Intraoperative fluroscopy was used in every case to confirm the position of the distal end of double-J stent.

Comparative analysis was performed between the two groups. Primary outcome was success of surgery. Secondary outcomes were other perioperative parameters. Categorical data were compared using chi-square or Fisher exact test. Continuous data were expressed as median with range or mean with standard deviation (SD). Continuous data were compared by Student *t* test or Mann-Whitney test as appropriate. Linear regression was used to investigate the trend of OT against increasing case experience. A *p*-value of < 0.05 was considered to be significant.

## Results

A total of 46 infants (LP = 22; RALP = 24) were included in this study. The medians of age and body weight were 6 months (2–12 months) and 8.0 kg (5.4–10 kg), respectively. There was no difference between the two groups in the patients' demographics and clinical characteristics at baseline (Table 1). No OP was performed for infants during the study period.

**Table 1:** Summary of the baseline characteristics of the two groups.

	**LP; *n* = 22**	**RALP; *n* = 24**	***p*-values**
Median age in months at the time of surgery (range)	6 (3–12)	5.5 (2–12)	0.97
Median body weight in kg at the time of surgery (range)	8.5 (5.4–10)	7.9 (5.7–10)	0.56
Gender: male/female	17/5	20/4	0.61
Laterality: left/right	16/6	13/11	0.19
Antenatal diagnosis	22/22	24/24	NA
Temporary PCN before surgery	3/22	6/24	0.33
Pre-operative imaging:			
APD in US	31 ± 12 mm	32 ± 12 mm	0.89
SRF in MAG3	44.8 ± 6.5%	45.6 ± 9.5%	0.74

*LP, laparoscopic pyeloplasty; RALP, robot-assisted laparoscopic pyeloplasty; US, ultrasound; APD, anteroposterior diameter; SRF, split renal function; PCN, percutaneous nephrostomy; NA, not applicable*.

All infants underwent LP or RALP successfully without conversion to open surgery or requirement of additional ports. None of the patients had intraoperative complications such as vascular or bowel injury, and none required blood transfusion. The estimated blood loss recorded was minimal with 5 ml or less.

**Figure 2:**
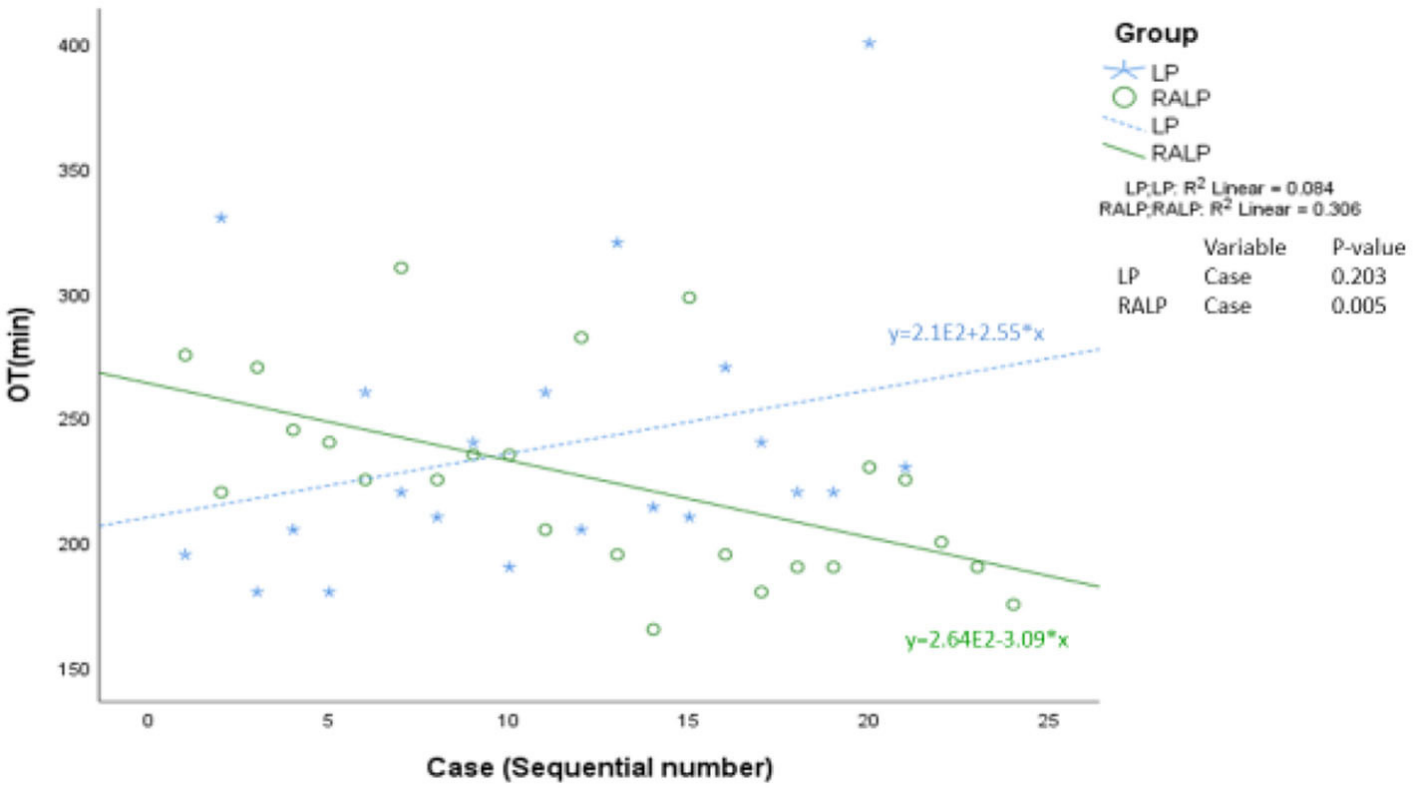
Linear regression analysis of plotting operative time against sequential case experience.

Table 2 summarized the perioperative parameters and post-pyeloplasty outcomes. OT was 242 min (SD = 59) in LP, compared with 225 min (SD = 39) of RALP (*p* = 0.25). Linear regression analysis showed a significant trend of decrease in OT with increasing case experience of RALP (*p* = 0.005) (Figure 2).

**Table 2:** Summary of the perioperative parameters and surgical success of the two groups.

	**LP; *n* = 22**	**RALP; *n* = 24**	***p*-values**
OT in minutes	242 ± 59	225 ± 3 9	0.25
Intraoperative cystoscopy	2/22	2/24	0.93
Aberrant crossing vessels	0/22	3/24	0.09
Participation of surgeon-in-training	7/22	12/24	0.21
Conversion to open or placement of additional ports	Nil	Nil	NA
Intraoperative complications or blood transfusion	Nil	Nil	NA
Mean number of doses of oral acetominophen per patient	3.8 ± 2.5	4.3 ± 3.2	0.55
Mean number of doses of intramuscular narcotics per patient	0.15 ± 0.50	0.04 ± 0.20	0.31
LOS in days	3.8 ± 1.3	3.0 ± 0.3	0.009
Post-operative complications (%)	4/22 (18)	3/24 (13)	0.59
Clavien Grade I – II	Prolonged ileus = 1 Stent-related UTI = 3	Stent-related UTI =2	
Clavien Grade IIIb		Proximal migration of Double-J stent = 1	
Operative success (%)	20/22 (91)	23/24 (96)	0.49
Mean follow-up in months	40 ± 16	23 ± 12	<0.001

*LP, laparoscopic pyeloplasty; RALP, robot-assisted laparoscopic pyeloplasty; OT, operative time; LOS, length of stay; UTI, urinary tract infection; NA, not applicable*.

No difference was noted in the post-operative analgesic requirement. RALP was associated with a shorter LOS of 3 days (SD = 0.3) compared with 3.8 days (SD = 1.3) of LP (*p* = 0.009). 4/22(18%) LP and 3/24(13%) RALP developed post-operative complications (*p* = 0.59). All but one of the complications were minor of Clavien grade I-II (prolonged ileus = 1; stent-related urinary tract infection = 5). The only Clavien grade III complication happened in the RALP group due to proximal migration of the double-J stent which was removed cystoscopically by a Fr 4 Amplatz Gooseneck snare catheter.

The success rates were 20/22(91%) in LP and 23/24(96%) in RALP (*p* = 0.49). The two failures in LP underwent redo-LP as they occurred before the introduction of RALP in our institution, and the single failure in RALP was treated by redo-RALP. All three redo-pyeloplasties were successful.

## Discussion

The existing data of minimally invasive pyeloplasty in infants are derived from case series (14, 15), comparative studies with OP (16, 17), and comparative studies with older children (18, 19). To the best of our knowledge, the present study is the first single-institution study to compare LP vs. RALP in infants. Others have reported their findings by comparing two cohorts of infants who underwent LP and RALP in two different institutions (20). Our study design of recruiting patients managed by the same surgeon may reduce the confounding effects caused by variations in surgical techniques, post-operative protocols, and in-patient practices which happened in multi-institutional studies (15, 20).

Our finding of 91% success rate of LP is similar to 92% reported by previous studies (14, 20) in infants. Concern has been raised whether the failure rate of LP could be higher in infants than in older children (21). A recent systematic review found an average success rate of 96.9% for LP in children (4). The authors, however, reported that there were very few studies targeted at infants (4). Failures of LP in infants may have been underreported, and the world-wide declining interest in LP has hampered further studies in infants for whom few surgeons perform LP (7).

The largest published series of RALP in infants was from a multi-institutional study which recruited 60 patients and reported 91% success rate (15). Two recent single-institution studies reported 93.8 and 94.1% success rates of RALP in 16 and 34 infants, respectively (18, 19). In both studies the authors did not note any difference in success rates between infants and older children (18, 19).

The latest meta-analysis performed by Taktak et al. included eight more studies comparing RALP vs. LP in pediatric populations (22) than the previous meta-analysis by Cundy et al. (1). The authors found a significantly higher success rate and shorter LOS in RALP than LP in children (22). Our findings of 96% success rate in RALP did not reach significant difference when compared with the 91% of LP. Further studies are warranted to investigate whether the potential superiority of pediatric RALP over LP in treatment success can be expanded to the infant population.

A bi-institutional study reported a significantly shorter LOS of RALP than LP in infants (1 vs. 7 days) (20). The authors, however, explained the finding by the difference in the healthcare systems and hospitalization polices of the two institutions where LP and RALP were separately performed (20). We found a statistically significant but small difference in LOS in favor of RALP(3 vs. 3.8 days). Our finding, however, needs to be interpreted with caution. The clinical significance of a difference in LOS of <1 day is questionable. Given the small number in either group, any outliers might have significant effect in the statistical analysis. Although all our study subjects were under the care of the same surgeon over the entire study period, we cannot exclude the possibility of a slight change in discharge criteria over time which might have disadvantaged the LP group in LOS. Nevertheless, it is our subjective experience that the robotic technology enhances the precision in tissue approximation and suturing, and thus has the potential to promote a faster recovery by allowing better tissue healing with less subclinical urine leakage.

It is debatable whether 5- or 8-mm instruments should be used in infant RALP. Use of 5-mm instruments allows a smaller incision at the cost of requiring a longer intracorporeal length for articulation due to its pulley system, which is the concern raised by some surgeons (8, 15). Proponents of 5-mm instruments, however, have reported the safety and similarly high success rates in infant and non-infant pediatric populations (18, 19). We have had no experience in using 5-mm instruments which are not supported by the current da Vinci Xi platform. Our findings of the post-operative analgesic requirement do not suggest any significant negative effects associated with the use of 8-mm ports in RALP when compared with LP using 5- and 3-mm ports. Nevertheless, we fully echo with others the need of the development of miniaturized robotic instruments specific for infants and small children (23).

Our OT of 225 min in infant RALP is much longer than the 115 and 144 min reported by master surgeons working at high-volume centers (16, 18), but similar to the 232 min reported by a multi-institutional study involving teaching hospitals with fellowship or residency training programs (15). Given our small case volume, we are still at a distance from achieving mastery in infant RALP. Our OT also included the time spent on undocking and redocking for fluoroscopy, and some cases involved training of surgeons who had not attained competency in pediatric RALP. We did not detect any difference in OT between the two groups of LP and RALP. However, the additional time spent on docking in RALP might suggest a faster procedure in RALP than LP, particularly during the intracorporeal suturing which the robotic platform alleviates much of the technical difficulty. The linear regression analysis demonstrated a significant trend of decrease in OT with increasing experience in RALP, and a trend of OT in favor of RALP after the first 10 cases. Given the two groups were comparable in other study variables, our finding suggests a faster learning curve of RALP than LP in infants.

Despite our long history of performing pediatric pyeloplasty by minimally invasive approach, our institution had only one surgeon left who was competent to perform LP in 2013. Since the adoption of RALP in 2014, there are currently three surgeons in our institution who are competent to perform pediatric RALP. We agree with others that the robotic technology offers the advantage of creating a more manageable learning curve for minimally invasive pyeloplasty, thus making it more accessible particularly to the infant population in which application of LP is even more challenging than older children (4).

We followed the technical tricks in infant RALP as described before with some modifications. Air leakage at the port site is more of a concern in infants than older children given the thin abdominal wall and its laxity in infants. We prevent air leakage by placing a purse-string suture to tighten the musculofascial defect around the camera port at the umbilicus and the suture was further tied onto the short rubber latex tube placed around the port to prevent it from accidentally slipping out. We did not anchor the two working ports to skin by sutures, and we made the incisions precisely such that the wounds were not any bigger than the trocars. Creating an adequate working space both intracorporeally and extracorporeally is critical to success in performing RALP in infants. Our ports positioning allows adequate distance to prevent trocar collision while avoiding the risk of bladder injury if the ports are all placed in midline as preferred by some surgeons (8, 15, 18). Elevation of the ports against the abdominal wall, and keeping a minimal depth of working ports inside the peritoneal cavity are both pivotal in maximizing the intracorporeal working space for small infants. It should be emphasized that excessive force in traction or grasping tissues may go unnoticed due to lack of tactile feedback of the robotic instruments, and extra caution must be exercised in infants whose tissues are more fragile than older children.

We acknowledged the limitations of our study including the retrospective nature, small case numbers over a long review period, lack of breakdown of OT, difference in follow-up periods, and lack of details of participation of surgeon-in-training. Patients were assigned the surgical approach chronologically without any randomization, and all the RALP cases were recruited after we had stopped performing LP. The prior acquisition of skills in LP may have given advantage in subsequent RALP. Our study findings did not allow estimation of the number of cases required to complete the learning phase of either technique. The generalizability of our data from a single institution is questionable, although some of the potential bias may be reduced by the standardized surgical techniques and management protocols. It was beyond the scope of the present study to investigate and compare the costs of the two procedures. The public healthcare service in our society is heavily subsidized by government such that it was almost free of charge for our patients' families whether the procedure was LP or RALP. There are no data in the billing system or from the finance department of our institution that we can retrieve to investigate the costs incurred from each surgical procedure. There is no question that it is a huge investment in purchasing a robotic platform, and the costs for maintenance and the disposable instruments are substantial. Previous single-institution studies have reported no difference in cost when RALP was compared with OP in infants (17), and when RALP was compared with LP in pediatric patients (24). At a national level, pediatric RALP was found to be associated with a higher cost than OP, and the relatively small number of pediatric pyeloplasty even in high-volume children's hospitals remained to be a limiting factor for reducing the cost of RALP (7). The robotic platform in our institution is shared among pediatric and adult patients. The high-volume adult robotic surgeries might give us an advantage in cost-effectiveness of performing pediatric RALP.

Given the paucity of data comparing the two minimally invasive modalities in infants, we believe our findings would contribute to the existing literature with addition evidence despite all the study limitations. Both LP and RALP are safe and effective modalities via a minimally invasive approach for correction of UPJ obstruction in infants. RALP appears to have superiority over LP in infants with its faster recovery, and a more manageable learning curve for skills acquisition. Our findings support the application of RALP across the entire pediatric population including infants.

## Data Availability Statement

The datasets presented in this article are not readily available because dataset not allowed to be accessed by outside of our institution. Requests to access the datasets should be directed to Yuk Him Tam, pyhtam@surgery.cuhk.edu.hk.

## Ethics Statement

The studies involving human participants were reviewed and approved by Joint CUHK-NTEC Clinical Research Ethics Committee. Written informed consent from the participants' legal guardian/next of kin was not required to participate in this study in accordance with the national legislation and the institutional requirements.

## Author Contributions

YW: study design, data collection and analysis, literature review, and draft manuscript. KP: data collection and analysis and literature review. YT: study design, literature review, and revised manuscript. All authors contributed to the article and approved the submitted version.

## Conflict of Interest

The authors declare that the research was conducted in the absence of any commercial or financial relationships that could be construed as a potential conflict of interest.
